# Circular RNA hsa_circ_0008896 accelerates atherosclerosis by promoting the proliferation, migration and invasion of vascular smooth muscle cells via hsa-miR-633/CDC20B (cell division cycle 20B) axis

**DOI:** 10.1080/21655979.2022.2039467

**Published:** 2022-02-25

**Authors:** Xumin Hou, Huangdong Dai, Yue Zheng

**Affiliations:** a Department of Cardiology, Shanghai Chest Hospital, Shanghai Jiao Tong University, Shanghai, China; b Department of Cardiovascular Surgery, Shanghai Chest Hospital, Shanghai Jiao Tong University, Shanghai, China

**Keywords:** Hsa_circ_0008896, atherosclerosis, hsa-miR-633, CDC20B

## Abstract

Circular RNAs, a class of circularly closed non-coding RNAs, play essential roles in the formation of atherosclerosis, which is a frequent cause of cardiovascular and cerebrovascular diseases. Although many circular RNAs are found to be involved in the progression of atherosclerosis, more circular RNA regulators still need to be identified, to improve the understanding of the regulatory networks of atherosclerosis. Here, we found that hsa_circ_0008896 was significantly up-regulated in both *in vitro* and *in vivo* atherosclerosis models, indicating hsa_circ_0008896 was involved in the progression of atherosclerosis. Further functional analyses confirmed that knockdown of hsa_circ_0008896 decreased proliferation, migration, and invasion of VSMCs. In addition, we conducted bioinformatics analysis and found that hsa-miR-633 could directly bind to hsa_circ_0008896, which was confirmed by RNA immune-precipitation (RIP) assays. Results of proliferation, migration, and invasion assays showed that hsa-miR-633 inhibitor reversed the si-circ_0008896 phenotypes, indicating that hsa_circ_0008896 functionally bound to hsa-miR-633. At last, combining bioinformatics and experimental analyses, we found the protein target of hsa_circ_0008896/hsa-miR-633, CDC20B (cell division cycle 20B). The expression level of CDC20B was regulated by hsa-miR-633, and knockdown of CDC20B decreased proliferation, migration, and invasion of VSMCs. Taken together, hsa_circ_0008896 regulated the expression of CDC20B by sponging hsa-miR-633, and then enhanced proliferation, migration, and invasion of VSMCs to promote the progression of atherosclerosis.

## Graphical Abstract

**Figure:**
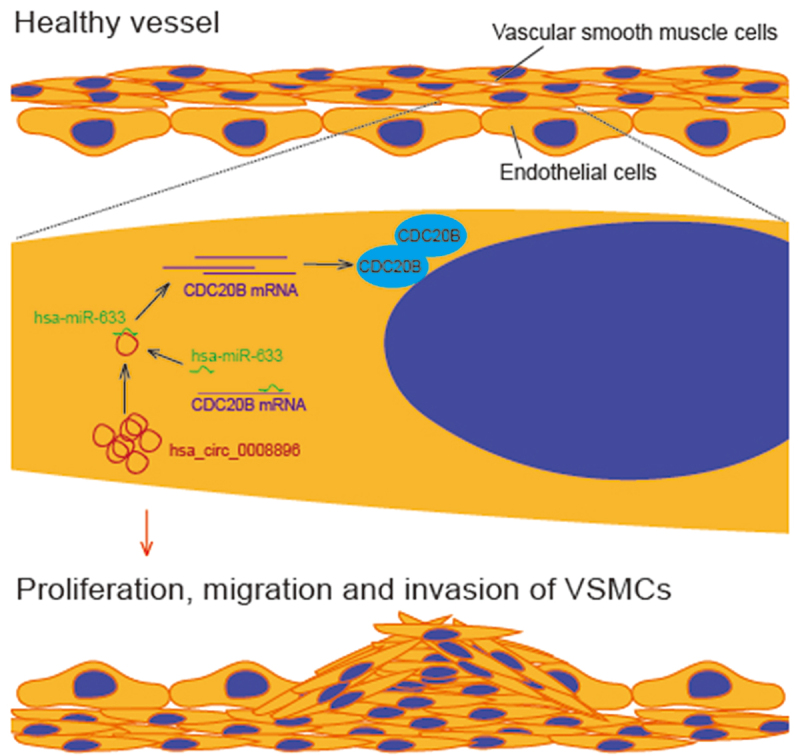

## Introduction

Atherosclerosis is a chronic progressive inflammatory disorder in which plaques form inside the arteries, and is the primary cause of morbidity and mortality in the world [1,2]. Atherosclerosis involves multiple pathological processes including endothelial dysfunction, vascular proliferation and the alternation of the extracellular matrix [3]. The proliferation of vascular smooth muscle cells (VSMCs) mainly contributes to the atherosclerosis process and is related to other cellular processes, including inflammation, lipid accumulation and apoptosis [4–6]. Epidemiological and experimental results suggest that increased level of oxidized low-density lipoprotein (ox-LDL) under oxidative stress and endothelium dysfunction are the main risk factors in atherosclerosis progression [7–9]. However, the precise molecular mechanism of atherosclerosis progression remains unclear, which hinders the development of efficiency therapies.

Non-coding RNAs, including long non-coding RNAs, circular RNAs and microRNAs, play important roles in regulating the proliferation and migration of VSMCs. For instance, miR-146b-3p represses the proliferation and migration of VSMCs induced by PDGF-BB, and long non-coding RNA PVT1/microRNA miR-3127-5p/NCK-associated protein 1-like axis regulates the proliferation of VSMCs [10,11]. Circular RNAs, a new class of single-stranded non-coding RNAs with closed-loop structures, are endogenous transcripts expressed in specific tissues and under specific pathological conditions [12–14]. Initially, circular RNAs are assumed to be transcriptional byproducts during RNA processing. It is now well known that many circular RNAs play important roles under certain pathological and physiological conditions [15]. For instance, circular RNAs play essential roles during the development of the cardiovascular system and in the pathological processes of coronary artery disease (CAD), a kind of heart abnormality [16]. Many circular RNAs were found to be regulators in the proliferation, migration and invasion of vascular smooth muscle cells (VSMCs) under atherosclerosis conditions. Circular RNA CHFR could facilitate VSMCs proliferation and migration by regulating miR-370/FOXO1/Cyclin D1 axis [17]. Circ_0029589 could regulate proliferation, migration, invasion, and apoptosis of ox-LDL treated VSMCs via miR-424-5p/IGF2 axis [18]. Circ_0029589 down-regulation could inhibit the proliferation, migration and invasion of VSMCs by modulating miR-214-3p/STIM1 axis [19]. Previous studies have shown that hsa_circ_0008896 was significantly up-regulated in oxidized low-density lipoprotein treated macrophages, indicating that hsa_circ_0008896 could function during the progression of atherosclerosis [20]. However, whether hsa_circ_0008896 functions under the pathological condition of atherosclerosis is still unclear.

In this study, we hypothesized that hsa_circ_0008896 could be a biomarker and regulator of atherosclerosis progression. The present study aims to identify the potential role of hsa_circ_0008896 in regulating the proliferation, migration and invasion of VSMCs. The purpose of this study is to discover novel molecular targets with the potential to be used therapeutically. Here, we reported that hsa_circ_0008896 was up-regulated in the atherosclerosis cellular and mice models, and hsa_circ_0008896 increased the expression of CDC20B by competitively binding to hsa-miR-633 to increase the proliferation, migration and invasion of VSMCs.

## Materials and methods

### Cell culture and the construction of atherosclerosis cellular model

Human vascular smooth muscle cells (VSMCs) were obtained from Bena Culture Collection, China (Suzhou, China). Human VSMCs were cultured using Dulbecco’s Modified Eagle Medium (Gibco, USA) containing 10% fetal bovine serum (Invitrogen, USA) and 1% penicillin/streptomycin (ST488, Beyotime, China). Human VSMCs were cultured in a 37°C incubator supplied with 5% CO_2_. To construct the cellular model of atherosclerosis, human VSMCs were stimulated with oxidized low density lipoprotein (ox-LDL, H7950, Solarbio, China) [21]. Si-NC, si-circ0008896, miR-633 mimics, miR-633 inhibitor, si-CDC20B and corresponding si-NC were provided by Guangzhou RiboBio Co., Ltd. Lipofectamine 2000 reagent (11,668,500, ThermoFisher, USA) was used for cell transfection.

### Quantitative RT-PCR (qRT-PCR)

Total RNA of VSMCs was extracted using the RNA Easy Fast Tissue/Cell Kit (DP451, TIANGEN, China) according to the manufacturer’s instructions. TaqMan™ MicroRNA Reverse Transcription Kit (4,366,596, ThermoFisher, USA) was used for the reverse transcription of microRNAs. The relative changes of gene expression were analyzed using 2^−ΔΔCt^ methods [22]. Primers used were as follows: hsa_circ_0008896: forward 5′-CATCCATTCCTCCTGCAATTTC-3′, reverse 5′-TAGTGTTTGTGCAGACTCCAT-3′; GAPDH: forward 5′-CGCTCTCTGCTCCTCCTGTTC-3′, reverse 5′-ATCCGTTGACTCCGACCTTCAC-3′; U6: forward 5′-AGCCCGCACTCAGAACATC-3′, reverse 5′-GCCACCAAGACAATCATCC-3′.

### RNase R digestion

VSMCs RNA was extracted as the previous description, and 10 μg total RNA was incubated with 50 U RNase R (RNR07250, Epicenter Technologies, USA) at 30°C for 15 min. After RNase R digestion, the digested products were reverse transcribed and the expression level of hsa_circ_0008896 was determined using qPCR methods as the previous description.

### Cell counting kit-8 (CCK-8) for the measurement of cell proliferation

The proliferation ability of VSMCs could be detected using cell counting kit-8 (CCK-8) [23]. 2 × 10^3^ VSMCs were seeded into a 96-well plate and then cultured for 2 hours. 10 μl CCK-8 solution (C0037, Beyotime, China) was pipetted into each well with VSMCs. Cells were incubated with CCK-8 solution for 2 hours, and the optional density (OD) value was determined using Multiskan FC with Incubator.

### Western blot assay

RIPA lysis buffer (P0013C, Beyotime, China) was used for the extraction of total VSMCs protein. The protein concentration was determined using BCA Protein Assay Kit (P0012S, Beyotime, China). Proteins were separated by electrophoresis and then transferred onto poly-vinylidene fluoride (PVDF) membrane. These membranes were incubated with 5% BSA for blocking for 1 hour, and then incubated in the primary antibody solution overnight. After washing, the PVDF membranes were incubated with the secondary antibody solution for 2 hours. The protein levels were detected with Pierce™ ECL Western blotting Substrate (32,109, ThermoFisher, USA) according to the instruction. Antibodies: Anti-Ki67 (1:1000, NBP2-54,791, Novus, USA); Anti-PCNA (1:800, NBP1-89,434, Novus, USA); Anti-GAPDH (1:1500, NB300-320, Novus, USA); Anti- CDC20B (1:500, 133,376-1-AP, Proteintech, USA).

### Clonogenic assay (colony formation assay)

10^3^ transfected VSMCs were seeded onto 6-well plates and treated with 50 μg/ml ox-LDL for 48 hours. VSMCs were incubated in the complete medium for 14 days. Then the cell colonies were fixed using 4% paraformaldehyde (PFA) for 2 hours at room temperature, and stained using 0.1% crystal violet for 2 hours. The colonies were imaged and counted using Image J software.

### Transwell assay

Cell migration and invasion were determined using transwell chambers (140,629, 8 µm pore size, ThermoFisher, USA). For the assessment of cell migration, VSMCs were re-suspended in FBS free culture medium, seeded in top chambers, and treated with 50 μg/ml ox-LDL. The culture medium with 10% FBS was pipetted to the well under the chamber. Subsequently, cells were cultured for 24 hours, stained with 0.1% crystal violet for 30 min, and imaged using a light microscope. For the assessment of cell invasion, the inserts were pre-coated with Matrigel (356,234, BD Biosciences, USA).

### Luciferase assay

The luciferase reporter plasmids containing hsa_circ_0008896 WT or Mut sequences were constructed and co-transfected into cells with hsa-miR-633. After incubation of 48 hours, Dual-Luciferase® Reporter Assay System (E1910, Promega, USA) was used for the detection of luciferase activities. Also, the luciferase reporter plasmids containing CDC20B 3ʹUTR WT or CDC20B 3ʹUTR Mut sequence were constructed and co-transfected into cells with hsa-miR-633, and the luciferase activities were measured using the Dual-Luciferase® Reporter Assay System.

### RNA immune-precipitation assay (RIP assay)

Hsa-miR-633 and corresponding control were transfected into VSMCs. To confirm whether hsa-miR-633 bound to hsa_circ_0008896 in an AGO2 manner, the anti-AGO2 antibody (MA5-23,515, ThemoFisher, USA) was incubated in the VSMCs lysate. Then, the expression level of hsa_circ_0008896 was determined by RT quantitative PCR.

### Statistical assessment

All experiments were repeated three times. All data were presented with mean ± SEM, and all statistical analyses were conducted with Prism GraphPad 7.0 using the Students’ *t* test. The significance was determined by *p* values. *P < 0.05, **P < 0.01, ***P < 0.001.

## Results

Accumulating evidence suggests that multiple circular RNAs participate in the progression of atherosclerosis; however, more non-coding RNAs involved in regulating atherosclerosis should be identified. In this study, we found that hsa_circ_0008896 was highly expressed in both *in vitro* and *in vivo* atherosclerosis models. Thus, we hypothesized hsa_circ_0008896 could be a biomarker and regulator of atherosclerosis progression. Hopefully, hsa_circ_0008896 could be identified as the regulator of atherosclerosis, and potential therapeutic target. Next, we explored the role of hsa_circ_0008896 in the proliferation, migration and invasion of VSMCs, and found that hsa_circ_0008896 indeed regulated the progression of atherosclerosis. Moreover, using bioinformatics analyses and further experiments, we found hsa_circ_0008896 promoted the expression of CDC20B via competitively binding to hsa-miR-633. Taken together, these results suggest that hsa_circ_0008896 could regulate the proliferation, migration and invasion of VSMCs via hsa-miR-633/CDC20B axis, and these molecules could be the potential therapeutic targets.

### Hsa_circ_0008896 was up-regulated in VSMCs stimulated with ox-LDL and the wire injured femoral artery

**Figure 1.**
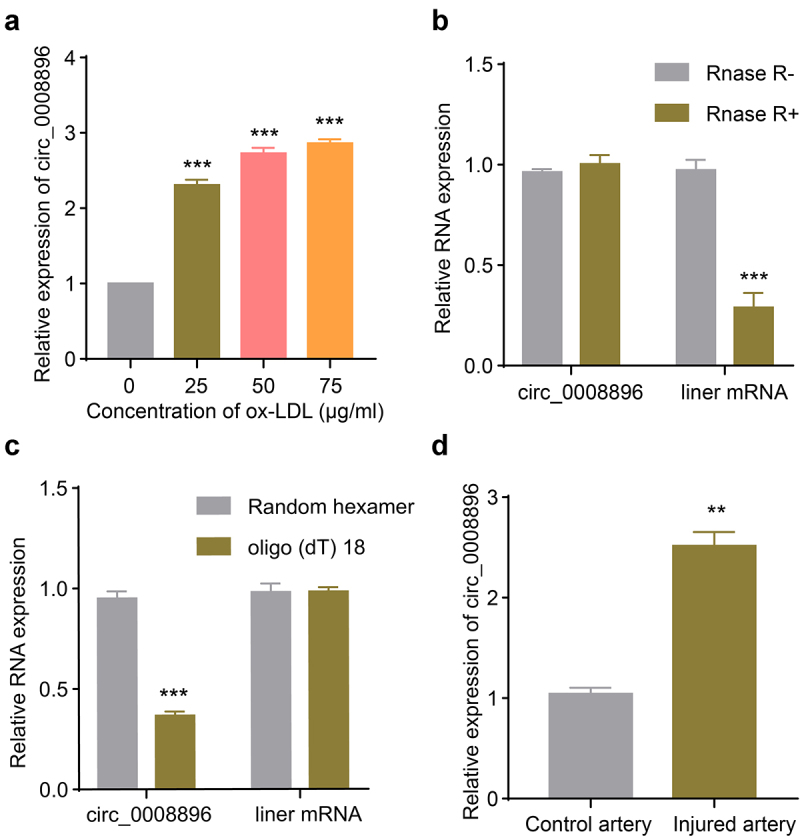
Hsa_circ_0008896 was up-regulated in VSMCs stimulated with ox-LDL and the wire injured femoral artery. (a) VSMCs were treated with different concentrations of ox-LDL. The expression level of hsa_circ_0008896 was then determined using quantitative PCR. (b) The expression levels of hsa_circ_0008896 and liner mRNA after Rnase R treatment were determined using quantitative PCR. (c) Hsa_circ_0008896 and liner mRNA amplified using random hexamer or oligo (dT) 18. The expression levels hsa_circ_0008896 and liner mRNA were detected using quantitative PCR. (d) The expression level of hsa_circ_0008896 in the wire injured femoral artery in mice was determined using quantitative PCR. **P < 0.01, ***P < 0.001.

A previous transcriptome study revealed that multiple circular RNAs were differentially expressed in the *in vitro* atherosclerosis cellular model stimulated with ox-LDL [20]. Hsa_circ_0008896 was up-regulated the most among circular RNAs, however, whether hsa_circ_0008896 played important role in the progression of atherosclerosis was still unclear. We first measured the expression level of circ_0008896 in the VSMC cellular model stimulated with ox-LDL. The results have shown that with the increasing concentration of ox-LDL treatment, circ_0008896 expression was up-regulated significantly (Figure 1a). We decided to use the concentration of 50 μg/ml ox-LDL treatment in the following experiments for the construction of the cellular atherosclerosis model. To confirm the circular feature of circ_0008896, RNase R treatment results revealed the stability of circ_0008896 (Figure 1b). We also employed random hexamer and oligo(dT)18 to amplify circ_0008896 and liner mRNA respectively, and the results confirmed the circular feature of circ_0008896 (Figure 1c). Finally, to further explore the expression level of hsa_circ_0008896 *in vivo*, we examined the expression of hsa_circ_0008896 using the femoral artery wire injury mice model. The results have shown that hsa_circ_0008896 expression was significantly up-regulated in the injured artery (Figure 1d).

### *Down-regulation of hsa_circ_0008896 inhibited proliferation, migration and invasion* in vitro

**Figure 2.**
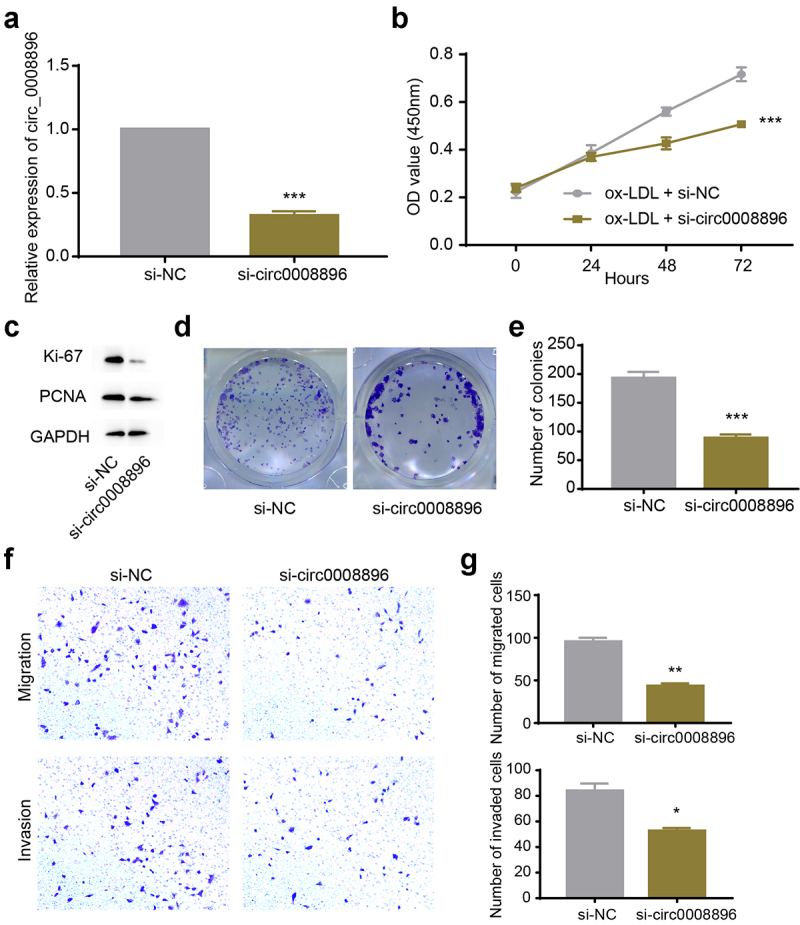
Down-regulation of hsa_circ_0008896 inhibited proliferation, migration and invasion *in vitro*. (a) The expression level of hsa_circ_0008896 after si-circ_0008896 transfection was detected using quantitative PCR. (b) CCK8 assay was conducted to measure cell viability after down-regulation of hsa_circ_0008896. (c) The expression levels of two proliferation markers, Ki67 and PCNA after down-regulation of hsa_circ_0008896 were detected using Western blotting. (d) Colony formation assay was conducted to examine proliferation ability after down-regulation of hsa_circ_0008896. (e) The quantification results of (d). (f) Transwell migration and invasion assays were conducted to examine the migration and invasion abilities of VSMCs after down-regulation of hsa_circ_0008896. (g) The quantification results of (f). *P < 0.05, **P < 0.01, ***P < 0.001.

Next, we conducted knockdown experiments to explore the function of circ_0008896. Firstly, we transfected si-circ_0008896 into VSMCs to efficiently knock down the expression level of circ_0008896 (Figure 2a). To measure explore whether the proliferation ability of VSMCs was regulated by circ_0008896, we conducted a CCK-8 assay, and found that si-circ_0008896 remarkably reduced the proliferation ability of VSMCs treated with ox-LDL (Figure 2b). Additionally, the levels of proliferation markers, including ki-67 and PCNA, were measured by Western blot. The results supported that si-circ_0008896 inhibited VSMCs proliferation (Figure 2c). To further assess the proliferation ability of VSMCs, a colony formation assay was conducted, and the results have shown that down-regulation of circ_0008896 significantly reduced the number of colonies, indicating that si-circ_0008896 indeed inhibited VSMCs proliferation (Figure 2d–e). Also, we observed the migration and invasion abilities of VSMCs after down-regulation of circ_0008896 by transwell assay. Transwell assay results indicated that down-regulation of circ_0008896 could efficiently reduce migration and invasion of VSMCs *in vitro* (Figure 2f–g).

### Hsa_circ_0008896 directly bound to hsa-miR-633

**Figure 3.**
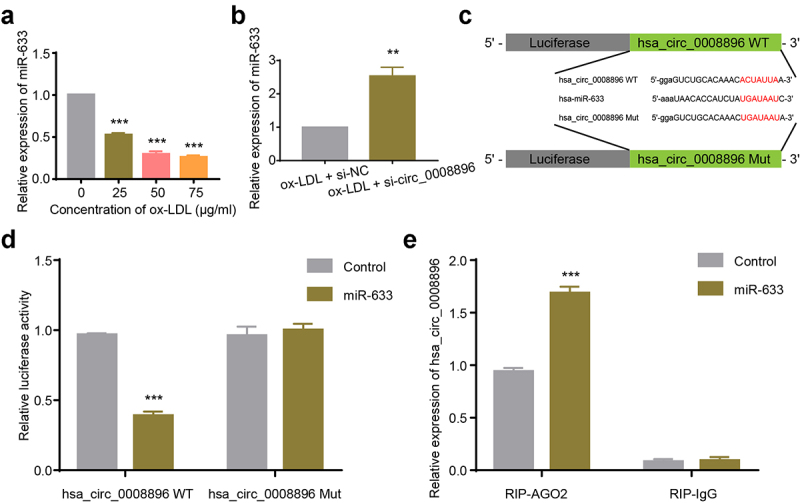
Hsa_circ_0008896 directly bound to hsa-miR-633. (a) VSMCs were treated with different concentrations of ox-LDL. The expression level of hsa-miR-633 was then determined using quantitative PCR. (b) The expression level of hsa-miR-633 in ox-LDL treated VSMCs transfected with si-circ-0008896 and its corresponding si-NC was determined using quantitative PCR. (c) The luciferase reporter plasmids were constructed as illustrated. (d) Relative luciferase activities after co-transfection of hsa_circ_0008896 WT and hsa-miR-633/Control, or hsa_circ_0008896 Mut and hsa-miR-633/Control were measured. (e) The expression levels of hsa_circ_0008896 in the hsa-miR-633+ RIP-AGO2 or hsa-miR-633+ RIP-IgG group was were determined using quantitative PCR. **P < 0.01, ***P < 0.001.

Circular RNAs could serve as sponges for microRNAs to play essential roles in diverse diseases. We then predicted the potential binding microRNAs of circ_0008896 using CircInteractome (https://circinteractome.nia.nih.gov) [24], and found that hsa-miR-633 potentially bound to hsa_circ_0008896. Firstly, we found that in the atherosclerosis cellular model, the level of hsa-miR-633 was decreased (Figure 3a). We then measured the level of hsa-miR-633 after down-regulation of circ_0008896, and the results have shown that si-circ_0008896 remarkably increased the expression of hsa-miR-633 (Figure 3b). For purpose of confirming the direct binding between circ_0008896 and hsa-miR-633, the luciferase reporter plasmids containing hsa_circ_0008896 WT and hsa_circ_0008896 Mut containing the hsa-miR-633 binding site were constructed as shown in Figure 3c. Co-transfection of hsa_circ_0008896 WT and hsa-miR-633 significantly reduced relative luciferase activity, while the luciferase activity in the hsa_circ_0008896 Mut and hsa-miR-633 co-transfection group remained unaffected, indicating that hsa_circ_0008896 could directly bind to hsa-miR-633 (Figure 3d). RNA immune-precipitation assay results suggested that hsa_circ_0008896 was enriched with the immune-precipitated AGO2 but not with IgG (Figure 3e).

### Hsa-miR-633 inhibitor could reverse the si-circ_0008896 phenotypes

**Figure 4.**
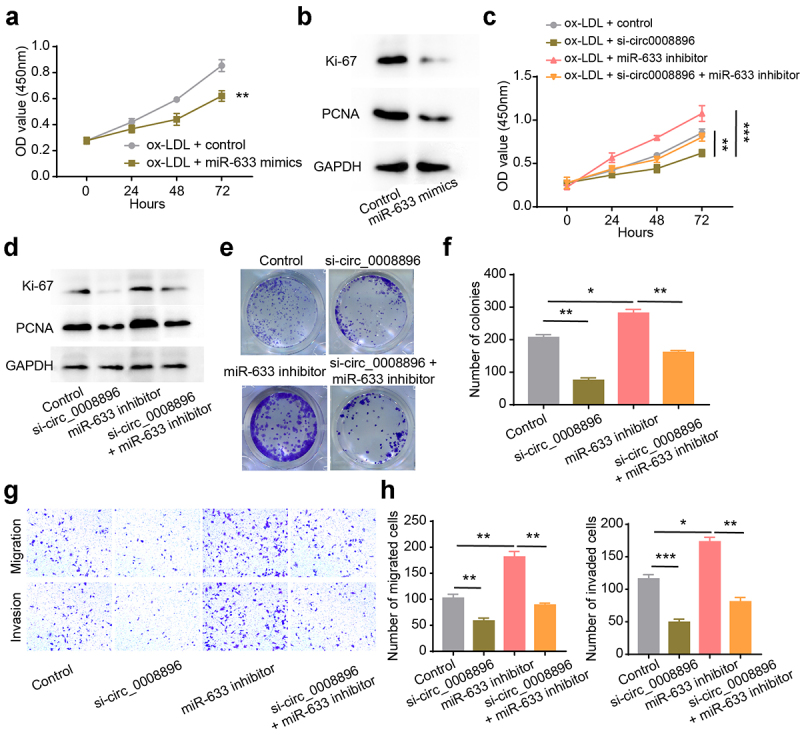
Hsa-miR-633 inhibitor could reverse the si-circ_0008896 phenotypes. (a) CCK8 assay was conducted to measure cell viability after up-regulation of hsa-miR-633. (b) The expression levels of two proliferation markers, Ki67 and PCNA after up-regulation of hsa-miR-633 were measured using Western blotting. (c) CCK8 assay was conducted to measure cell viability after the treatment of si-circ0008896, hsa-miR-633 inhibitor and si-circ0008896+ hsa-miR-633 inhibitor. (d) The expression levels of two proliferation markers, Ki67 and PCNA after the treatment of si-circ0008896, hsa-miR-633 inhibitor and si-circ0008896+ hsa-miR-633 inhibitor were measured using Western blotting. (e) Colony formation assay was conducted to examine proliferation ability number of colonies after the treatment of si-circ0008896, hsa-miR-633 inhibitor and si-circ0008896+ hsa-miR-633 inhibitor. (f) Quantification results of (e). (g) Transwell migration and invasion assays were conducted to examine the migration and invasion abilities of VSMCs after the treatment of si-circ0008896, hsa-miR-633 inhibitor and si-circ0008896+ hsa-miR-633 inhibitor. (h) Quantification results of (g). *P < 0.05, **P < 0.01, ***P < 0.001.

To explore whether hsa-miR-633 functionally correlated with circ_0008896, we up-regulated hsa-miR-633 level using hsa-miR-633 mimics. The results have shown that after up-regulation of hsa-miR-633, the proliferation ability of VSMCs was significantly decreased (Figure 4a–b). And hsa-miR-633 inhibitor increased the OD values and the expression levels of proliferation markers (Figure 4c–d). Additionally, we found that hsa-miR-633 inhibitor could reverse the decreased proliferation ability of VSMCs caused by circ_0008896 knockdown by conducting CCK-8 and colony formation assays and measuring the expression levels of proliferation markers (Figure 4c–f). Next, transwell assay results have shown that hsa-miR-633 inhibitor could also reverse the decreased migration and invasion abilities of VSMCs caused by circ_0008896 knockdown (Figure 4g–h). Taken together, the hsa-miR-633 inhibitor could reverse the si-circ_0008896 phenotypes, indicating that hsa-miR-633 functioned as the downstream of circ_0008896.

### CDC20B was the protein effector regulated by hsa-miR-633

**Figure 5.**
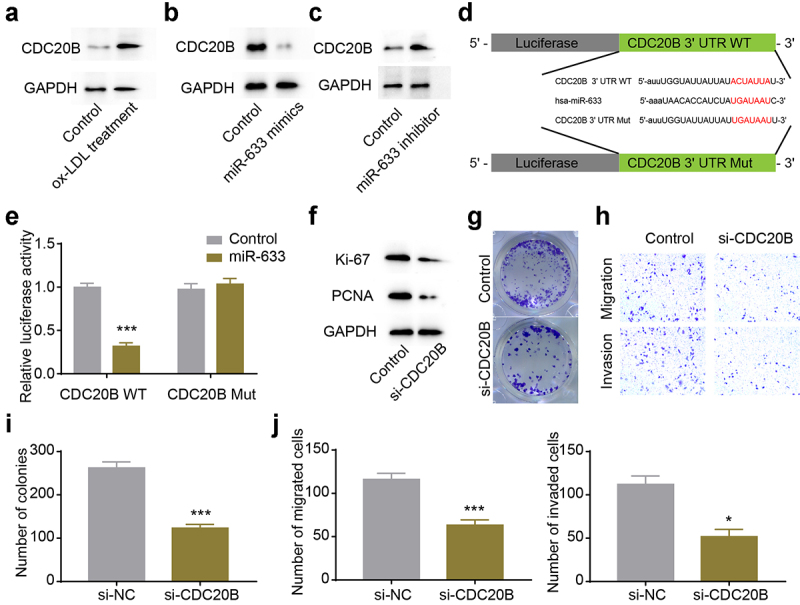
CDC20B was the protein effector regulated by hsa-miR-633. (a) CDC20B level was up-regulated after ox-LDL treatment revealed by Western blotting. (b) CDC20B level was decreased after the treatment of hsa-miR-633 mimics revealed by Western blotting. (c) CDC20B level was increased after the treatment of hsa-miR-633 inhibitor revealed by Western blotting. (d) The luciferase reporter plasmids were constructed as illustrated. (e) Relative luciferase activities after co-transfection CDC20B WT and hsa-miR-633/Control, or CDC20B Mut and hsa-miR-633/Control were measured. (f) The expression levels of two proliferation markers, Ki67 and PCNA after the treatment of si-CDC20B were measured using Western blotting. (g) Colony formation assay was conducted to examine the proliferation abilityafter the treatment of si-CDC20B. (h) Transwell migration and invasion assays were conducted to examine the migration and invasion abilities of VSMCs after the treatment of si-CDC20B. (i) Quantification results of (g). (j) Quantification results of (h). *P < 0.05, **P < 0.01, ***P < 0.001.

MicroRNAs could affect the stability and translation of RNAs at the post-transcriptional level by directly binding to the 3ʹUTR of mRNAs [25]. To identify the potential protein effector regulated by hsa-miR-633, we conducted bioinformatics analysis using TargetScan [26], and found CDC20B 3ʹUTR contained a potential binding site for hsa-miR-633. Firstly, we examined the CDC20B level in our cellular atherosclerosis model stimulated with ox-LDL, and found that CDC20B expression increased in the cellular atherosclerosis model (Figure 5a). Subsequently, we up- or down-regulated hsa-miR-633, and found that CDC20B expression was negatively correlated with hsa-miR-633 level, suggesting hsa-miR-633 could regulate the expression of CDC20B (Figure 5b–c). Next, the luciferase reporter plasmids were constructed as illustrated (Figure 5d), and the decreased relative luciferase activity in the CDC20B 3ʹUTR and hsa-miR-633 group indicated that hsa-miR-633 could directly bind to the 3ʹUTR of CDC20B (Figure 5e). In order to To confirm that CDC20B was the protein effector regulated by circ_0008896 and hsa-miR-633, we down-regulated CDC20B using si-CDC20B in VSMCs. The proliferation ability of VSMCs was significantly decreased after CDC20B knockdown revealed by the decreased expression levels of proliferation markers and the decreased number of colonies in the colony formation assay (Figure 5f, g and i). Also, the migration and invasion abilities of VSMCs were remarkably decreased in the si-CDC20B group revealed by the transwell assay (Figure 5h and j). Taken together, the expression level of CDC20B was regulated by hsa-miR-633, and CDC20B functioned as the downstream protein effector of circ_0008896 and hsa-miR-633.

## Discussion

In the pathological progress of atherosclerosis, endothelial cells, leukocytes, and intimal smooth muscle cells are all involved [27], especially vascular smooth muscle cells (VSMCs), which is a major cell type present at all stages of the atherosclerotic plaque [28]. In this study, we demonstrated that hsa_circ_0008896 regulated the hsa-miR-633/CDC20B axis to enhance proliferation, migration, and invasion of VSMCs, contributing to the progression of atherosclerosis. Our findings may provide new therapeutic targets for the development of atherosclerosis drugs.

Increasing studies have confirmed that circular RNAs, a kind of endogenous non-coding RNAs, play essential roles during multiple pathological processes with cell type and tissue specificities, indicating the potential biological functions of circular RNAs [29]. Previous studies have identified numerous circular RNAs involved in the progression of atherosclerosis. For instance, antisense noncoding RNA in the INK4 locus (ANRIL), the noncoding RNA in chromosome 9q21, was strongly associated with atherosclerosis [30]. As we all know, after the vascular wall injuries, VSMCs could proliferate and migrate efficiently to the tissue repair [31]. Abnormal proliferation, migration and invasion of VSMCs contribute to the pathological process of atherosclerosis. For instance, circWDR77 was highly expressed in VSMCs treated with high glucose, and circWDR77 inhibition decreased the proliferation and migration of VSMCs by miR-124/FGF2 axis [32]. In our study, we identified hsa-circ_0008896 as a highly expressed atherosclerosis marker in both *in vitro* and *in vivo* atherosclerosis models, suggesting that hsa-circ_0008896 may serve as an atherosclerosis marker in the early diagnosis.

Nowadays, it is widely accepted that circular RNAs are involved in competitive regulatory interactions, known as competing endogenous RNA (ceRNA) networks [25,33,34]. Circular RNAs can act as microRNA sponges through the ceRNA manner to regulate the expression of downstream genes. In our study, we searched for the binding partner of hsa-circ_0008896 using both bioinformatics and experimental methods, and confirmed that hsa-miR-633 functioned as the binding partner of hsa-circ_0008896.

In order to identify the gene regulated hsa-circ_0008896, we then conducted bioinformatics search and found CDC20B could be the potential target. We detected the expression level of CDC20B in the cellular model of atherosclerosis, and results showed CDC20B was up-regulated in VSMCs treated with ox-LDL, indicating CDC20B expression level was regulated by hsa-circ_0008896. CDC20B, a member of the cell division cycle 20 (CDC20) family, is required during the nuclear movement prior to anaphase in the cell cycle [35]. Aberrant expression of CDC20 is confirmed to be related to malignant progression of various cancers, and down-regulation of CDC20 suppresses the migration of pancreatic cancer cells [36]. Recently, high expression of CDC20B was found to be associated with a low survival rate of ovarian cancer, suggesting that CDC20B might be an oncogene regulating the proliferation of cancer cells [37]. In our study, we found the expression level of CDC20B was tightly controlled by hsa-miR-633. Additional functional assays also confirmed that CDC20B was the functional target of hsa-miR-633.

The regulated target of hsa-circ_0008896, CDC20B, participates in the nuclear movement prior to anaphase in the cell cycle [35], indicating that cell cycle associated proteins may be key players in regulating the proliferation of VSMCs in the pathological process of atherosclerosis. Indeed, a recent study has found that Salidroside, one of the active ingredients of *Rhodiolacrenulata*, prevented ox LDL treated endothelial cell senescence by promoting cell cycle progression by the phosphorylation of the retinoblastoma (Rb) protein [38]. Accumulating evidence suggest there are complex interactions between atherosclerosis and cell cycle progression of multiple cell types. Cell cycle is the main factor leading to cell proliferation. However, cell death is also closely connected to cell cycle. In the future, we may further explore the role of hsa-circ_0008896 in the regulation of VSMCs cell cycle and apoptosis.

However, numerous issues need to be further clarified. First, data in our study were collected using *in vitro* cellular model and *in vivo* animal model. The role of hsa-circ_0008896 in atherosclerosis should be further confirmed using RNA sequencing data from clinical atherosclerosis samples. Second, we need to testify the safety of manipulation of hsa-circ_0008896 level *in vivo* using mice before further clinical trials. Third, cell cycle is precisely controlled via a network of many genes and non-coding RNAs. The role of hsa-circ_0008896 in the regulatory network should be further studied.

## Conclusion

Hsa_circ_0008896, which was highly expressed in both *in vitro* and *in vivo* atherosclerosis, increased the expression of CDC20B via binding to hsa-miR-633. Our results identify the role of hsa_circ_0008896 in the proliferation, migration and invasion of VSMCs, and suggest hsa-miR-633 and CDC20B could be potential therapeutic targets; however, further clinical data should be analyzed to confirm these findings.

## Author contribution

Y Z conceived and designed the analysis; XM H and HD D collected the data; XM H and HD D performed the analysis; XM Hand HD D contributed equally to this work; Y Z wrote the manuscript with inputs from all authors.

## Data Availability

All data generated or analyzed during this study are included in the manuscript.
